# TUG1, SPRY4-IT1, and HULC as valuable prognostic biomarkers of survival in cancer: A PRISMA-compliant meta-analysis

**DOI:** 10.1097/MD.0000000000008583

**Published:** 2017-11-17

**Authors:** Yucheng Zhong, Zhicong Chen, Shuyuan Guo, Xinhui Liao, Haibiao Xie, Yien Zheng, Bin Cai, Peixian Huang, Yuhan Liu, Qun Zhou, Yuchen Liu, Weiren Huang

**Affiliations:** a Key Laboratory of Medical Reprogramming Technology, Shenzhen Second People's Hospital, The First Affiliated Hospital of Shenzhen University, Shenzhen; b Department of Urology, Peking University First Hospital, The Institute of Urology, Peking University, National Urological Cancer Centre, Beijing; c Shantou University Medical College, Shantou, China.

**Keywords:** HULC, LncRNA, meta-analysis, prognosis, SPRY4-IT1, TUG1

## Abstract

**Background:**

Long noncoding RNAs (LncRNAs) are involved in the development and progression of various cancers. Accumulating evidences indicated that expression of lncRNAs was related to the prognosis of tumors.

**Methods:**

Here, 3 well-known lncRNAs associated with cancer were gathered to prove the potential role of lncRNAs as novel predictors of survival in human cancer. This meta-analysis collected all eligible studies about TUG1, SPRY4-IT1, and HULC and explored the relationship between lncRNAs expression and lymph node metastasis (LNM) or overall survival (OS). A comprehensive, computerized literature search was undertaken by using PubMed, EMBASE, Cochrane Library, and Web of Science (up to October 10, 2017). Strength of association between 3 lncRNAs and cancer prognosis was assessed by computing the hazard ratios (HR) with its corresponding 95% confidence interval (CI). According to the inclusion and exclusion criteria, respectively, 10, 9, and 7 studies of 3 lncRNAs were included in this meta-analysis.

**Results:**

In the current meta-analysis, it could be concluded that the expression of these 3 lncRNAs in tumor tissues is not a direct evidence of LNM. In general, there was a significant negative correlation between TUG1 levels and OS time (pooled HR 1.54, 95% CI 1.06–2.24), SPRY4-IT1 levels and OS time (pooled HR 2.12, 95% CI 1.58–2.86) and HULC levels and OS time (pooled HR 2.10, 95% CI 1.18–3.73). It could be revealed from the result that high level expression of these 3 lncRNAs might be correlated with a bad prognosis.

**Conclusions:**

In conclusion, the current meta-analysis demonstrated that TUG1, SPRY4-IT1, and HULC might serve as a moderate predictor of survival in human cancer.

## Abstract 2

Supplemental Digital Content is available in the text

## 1 Introduction

Cancer has been a serious public health problem world widely since the beginning of the 20th century, the overall incidence and mortality rate are on the rise.^[1]^ Cancer incidence and mortality have been increasing in China, making cancer the leading cause of death since 2010 and a major public health problem in the country.^[2]^ As known to us that, in the early stage of tumors without lymph node metastasis (LNM) or distant metastasis, some therapies such as chemotherapy, radiotherapy, and surgery, are effective. However, because of their gradual progression and nonspecific symptoms, most cancers are usually diagnosed in advanced stage.

Furthermore, the prognosis of tumor cannot be fully interpreted by the current cancer staging. The 2 types of commonly used cancer staging methods are as follows. One is Clinical staging: I, II, III, IV, based on a large number of case studies and follow-up results analysis, grouped by the survival rate of patients staging. The other is the TNM staging method. TNM staging determines the local tumor size (T), whether there is regional lymph node metastasis and extent of transfer (N), or distant metastasis (M). TNM stages of tumor lesions are described in detail.

As the development of gene sequencing technology and indepth study of cancer biology, these 2 stage standards have encountered enormous challenges. The more we study the biology of cancer, the more things should be updated, like staging standard and diagnostic methods of cancer. Therefore, early detection of tumors, especially, finding a novel molecular cancer marker is both important and necessary for cancer patients to have timely treatment. Biomarker is indispensable to predict LNM and prognosis by observing the progression of cancers and estimating the prognosis.

Because of the rapid development of second-generation sequencing technology,^[3,4]^ long noncoding RNAs (lncRNAs), defined as noncoding transcripts 200 nucleotides longer in length, have been involved in the development of various human diseases, particularly in cancers.^[5–9]^ LncRNAs also play crucial regulatory roles in different cellular processes, such as gene regulation, posttranslational processing, and tumor genesis.^[10]^

Meanwhile, it has been shown that many lncRNAs can function as oncogenes or tumor suppressors.^[11–15]^ LncRNA-taurine up-regulated1 (TUG1) was initially detected in a genomic screen for genes upregulated in response to taurine treatment in developing mouse retinal cells.^[16]^ SPRY4 intronic transcript 1 (SPRY4-IT1), which was observed in melanoma, could modulate cell proliferation, cell apoptosis, and invasion.^[17]^ Hepatocellular carcinoma upregulated long noncoding RNA (HULC), located in Chromosome 6p24.3, first found in hepatocellular carcinoma (HCC) patients, has been highly associated with cancers’ diagnosis.^[18]^ TUG1, SPRY4-IT1, and HULC were involved in the occurrence and development of various cancers including muscle-invasive bladder cancer (MIBC),^[19]^ esophageal squamous cell carcinoma (ESCC),^[20]^ glioma (GLA),^[21]^ osteosarcoma (OSA),^[22]^ colorectal cancer (CRC),^[23]^ gastric cancer (GC),^[16]^ renal cell carcinoma (RCC),^[24]^ and nonsmall cell lung cancer (NSCLC).^[25,26]^ Their aberrant expressions were closely linked to the clinical pathological characteristics, such as lymph node metastasis, distant metastasis, and overall survival. Therefore, the lncRNA-TUG1, SPRY4-IT1, and HULC may function as potential markers in predicting the prognosis of patients with various kinds of cancer.

However, major limitation also has been revealed for the insufficient size of samples and inconsistent results. Therefore, a systematic review and meta-analysis has been carried out to explore the expression of these 3 well-known lncRNAs (TUG1, SPRY4-IT1, and HULC) and lymph node metastasis and the overall survival to prove these 3 lncRNAS might serve as biomarkers in cancer prognosis and diagnosis.

## 2 Materials and methods

### 2.1 Meta-analysis

This report is strictly in accordance with the PRISMA guidelines.^[27]^ All analyses were based on previous published studies, thus no ethical approval and patient consent are required.

### 2.2 Search strategy

Electronic databases PubMed, EMBASE, Cochrane Library, and Web of Science were systematically performed by using “lncRNA-TUG1 or TUG1 and cancer or tumor or carcinoma,” “lncRNA-SPRY4-IT1 or SPRY4-IT1 and cancer or tumor or carcinoma,” “lncRNA-HULC or HULC and cancer or tumor or carcinoma” as keywords separately, to identify potentially relevant studies. The latest update of searching was on October 10, 2017.

### 2.3 Inclusion and exclusion criteria

Inclusion criteria of the studies are as follows: Articles associating with these 3 well-known lncRNAs (TUG1, SPRY4-IT1, and HULC) expression and prognosis of the patients should be investigated. Patients were grouped according to the expression levels of lncRNAs, which were measured in primary tumor tissues. Related clinical pathological characteristics were reported, including lymph node metastasis. Clinical outcomes including overall survival were reported. Eligible articles should contain information on hazard ratios (HR) and corresponding 95% confidence intervals (CI), even if there is no explicit HR in the text, a survival curve should be contained. It is available for the full text. Exclusion criteria are as follows: duplicate publications; nonhuman study or noncomparative or irrelevant; reviews, case reports, letters, editorials, and expert opinions; studies were not grouped according to the expression level of lncRNAs; and studies without available data (explicit HR or a survival curve).

### 2.4 Risk of bias assessment

The biased risk assessment of each eligible study was based on the basis for assessing the internal validity of the prognostic article^[28,29]^ and the recommendations on the biomarker research report.^[30,31]^

### 2.5 Data extraction

According to our criteria, 2 authors (BC and PXH) independently assessed the qualification of the retrieved articles being searched. Any doubt was committed by consensus with YEZ. About data extraction tools, firstly, a standardized Microsoft Excel table has been adopted according to the CHARMS checklist^[32]^: first author, publication date, country of origin, tumor type, detected sample size, detection method of these 3 well-known lncRNAs (TUG1, SPRY4-IT1, and HULC) expression levels, cut-off values, number of high lncRNAs expression group and low lncRNAs expression group, number of patients with lymph node metastasis, survival analysis, multivariate analysis, follow-up period, HR, and corresponding 95% CIs for overall survival (OS). If only Kaplan–Meier curves were available, data from the graphical survival plots have been extracted and the HRs have been estimated. The process of data extraction was standardized by 3 authors (YCZ, ZCC, and SYG) and 1 author (YCL) independently intervened to monitor the whole process and achieved consensus in the case of disagreement. All calculations mentioned above were based on the methods illustrated by Parmar et al^[33]^ and Tierney et al^[34]^

According to the inclusion and exclusion criteria, data were extracted independently by 3 authors (YCZ, ZCC, and SYG). Disagreements were resolved by 2 investigators (YCL, WRH) through discussions. The extracted information for each eligible study included: first author, publication date, country of origin, tumor type, detected sample size, detection method of these 3 well-known lncRNAs (TUG1, SPRY4-IT1, and HULC) expression levels, cut-off values, number of high lncRNAs expression group and low lncRNAs expression group, number of patients with lymph node metastasis, survival analysis, multivariate analysis, follow-up period, HR, and corresponding 95% CIs for OS. If only Kaplan–Meier curves were available, we extracted data from the graphical survival plots and estimated the HRs. All calculations mentioned above were based on the methods illustrated by Parmar et al^[33]^ and Tierney et al.^[34]^

### 2.6 Statistical methods

All the statistical analyses were performed with Stata 12.0. The result of the odds ratios was calculated according to the bivariate variables of LNM results. Data of pooled HR were extracted from the qualified studies; the log HR and standard error (SE) were used for combination of the survival results.^[34]^ To evaluate the heterogeneity of the eligible studies, pooled HR were executed by using *I*^*2*^ statistics in this meta-analysis.^[35]^ Subgroup analysis was performed on the basis of the expression of lncRNAs. For studies evaluating the association between TUG1 expression and prognosis, the subgroup analysis was adopted to discuss the effects of high and low expression of TUG1 in diverse cancers respectively. HULC also uses the above method for subgroup analysis. The random-effects model was employed for the meta-analysis. Sensitivity analyses are important components of meta-analyses to assess the sensitivity of heterogeneity measures to exclusion of studies, and sensible in particular to define a ‘desired threshold’ in terms of the *I*^*2*^ or tau-square statistic. The potential publication bias was measured through the Egger test. *P* < .05 were examined to be statistically significant.

## 3 Results

### 3.1 Selection of studies

**Figure 1:**
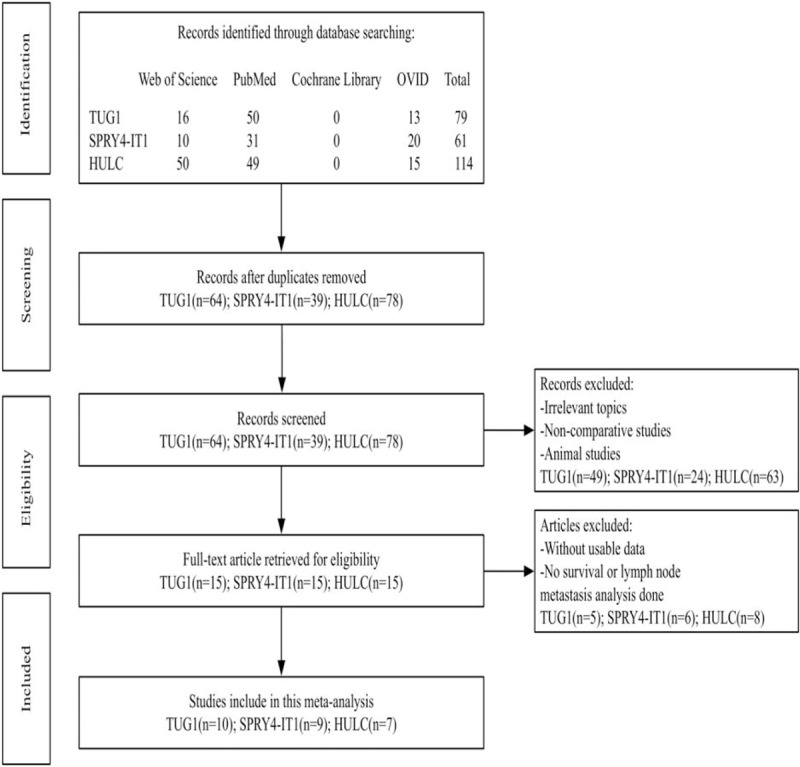
Flowchart presenting the steps of literature search and selection.

A total of 79 (TUG1), 61 (SPRY4-IT1), and 114 (HULC) published records were retrieved in our preliminary search by looking up the keywords. Moreover, 15, 22, and 36 duplicate references subsequently were excluded. After the title and abstract being screened, 49, 24, and 63 irrelevant references were further excluded. Upon further review of the full articles, a total of 26 publications addressing the relationship between lncRNA and cancer LNM or OS were found to meet all of the inclusion criteria and used for data extraction. Finally, this current meta-analysis was conducted for the remaining 10 (TUG1), 9 (SPRY4-IT1), and 7 (HULC) studies. (Fig. 1)

### 3.2 Characteristics of eligible studies

The eligible studies were published from 2014 to 2017. In the total 26 included studies, 24 were from China and the other 2 were from Czech Republic and Brazil. The types of cancers in the included studies were as follows: muscle-invasive bladder cancer, nonsmall cell lung cancer, glioma, osteosarcoma, colorectal cancer, gastric cancer, renal cell carcinoma, urothelial carcinoma of the bladder, esophageal squamous cell carcinoma, ovarian cancer, and cervical cancer. All the detected samples were tissues or frozen tissues from patients without chemotherapy or radiotherapy before surgery. The expression of TUG1, SPRY4-IT1, and HULC was measured by qRT-PCR and normalized to GAPDH or β-actin. In all the studies, the patients were divided into 2 groups: high and low expression of lncRNAs. All the diagnoses of lymph node metastasis were based on pathology. Among the 26 included studies, not all studies were examined with both OS and LNM. All the studies were of high quality (Table 1)^[19–26,36–50]^ as confirmed by the Newcastle-Ottawa Scale (NOS) in Table 2.^[19–26,36–50]^

**Table 1:**
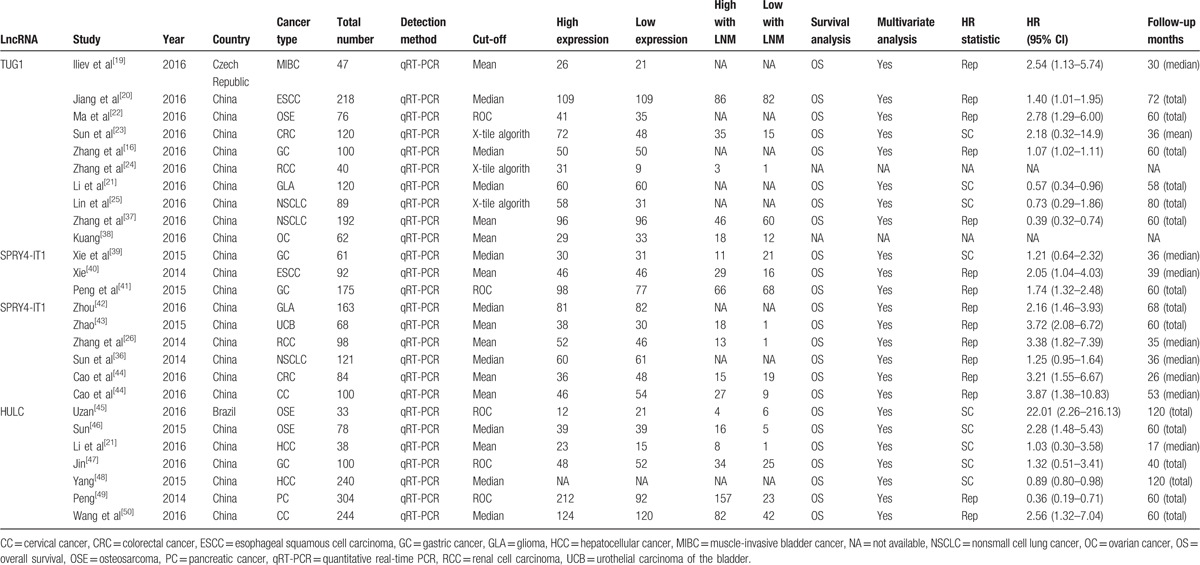
Characteristics of studies in this meta-analysis.

**Table 2:**
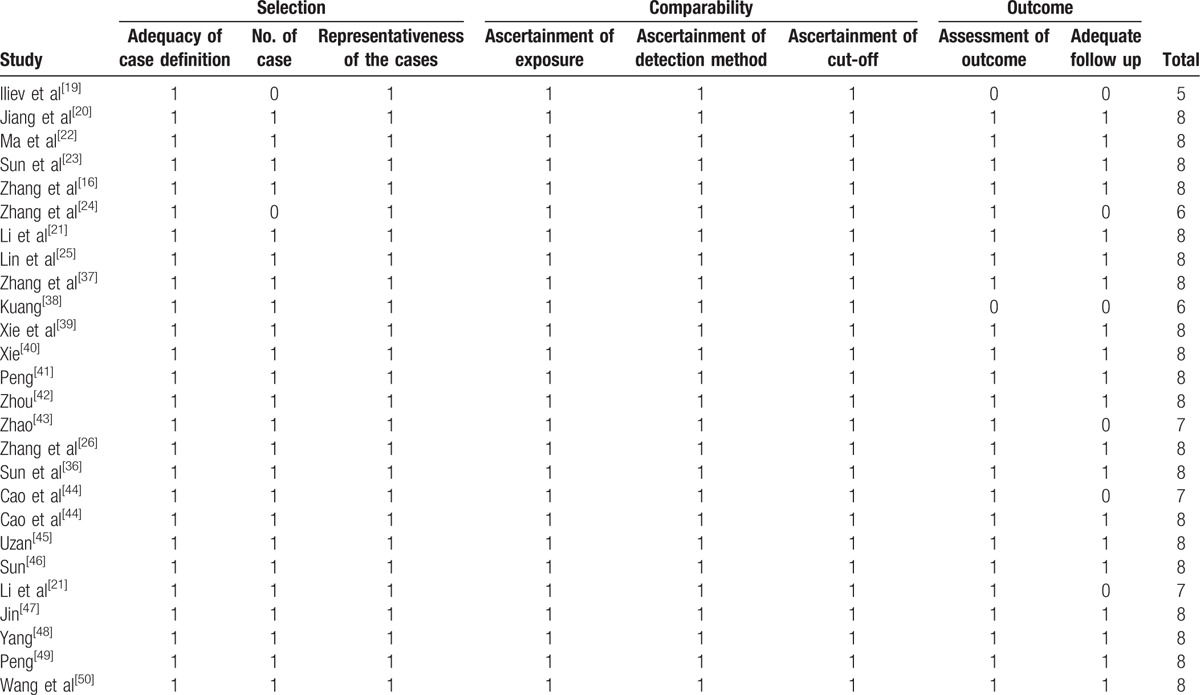
Quality assessment of eligible studies (Newcastle-Ottawa Scale).

### 3.3 Meta-analysis

#### 3.3.1 Association between 3 lncRNAs and LNM

**Figure 2:**
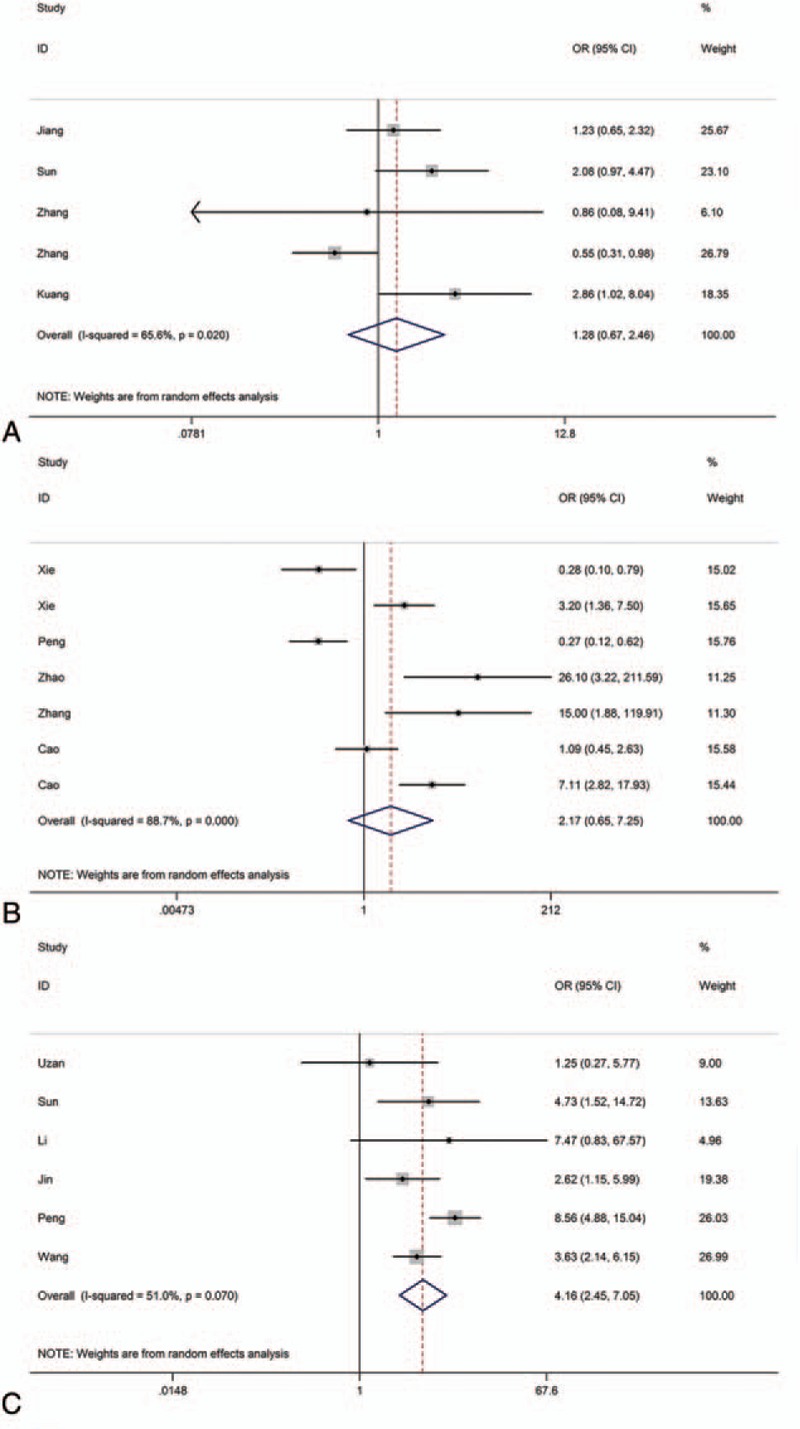
Forest plot of the correlation between the expression level of 3 lncRNAs expression levels and LNM in different cancer patients.

Five studies reporting a total of 632 patients with LNM were included on the basis of different TUG1 expression patterns. The random-effects model was expected to be adopted. Analysis showed that the OR of 1.28 with 95% CI 0.67–2.46 (*P* = .459), which meant the expression of TUG1 might not be a direct predictor of LNM (Fig. 2 A).

Seven studies reporting a total of 678 patients with LNM were included on the basis of different SPRY4-IT1 expression patterns. The random-effects model was expected to be adopted. Analysis showed the OR of 2.17 with 95% CI (0.65–7.25) (*P* = .210), which revealed that the expression of SPRY4-IT1 might not be an available predictor of LNM (Fig. 2 B).

Six studies reporting a total of 797 patients with LNM were included on the basis of different HULC expression patterns. The random-effects model was adopted. Analysis showed the OR of 4.16 with 95% CI (2.45–7.05), that is the expression of HULC does have a positive influence on LNM. HULC might serve as a direct predictor of LNM. (Fig. 2C)

#### 3.3.2 Association between 3 lncRNAs and OS

**Figure 3:**
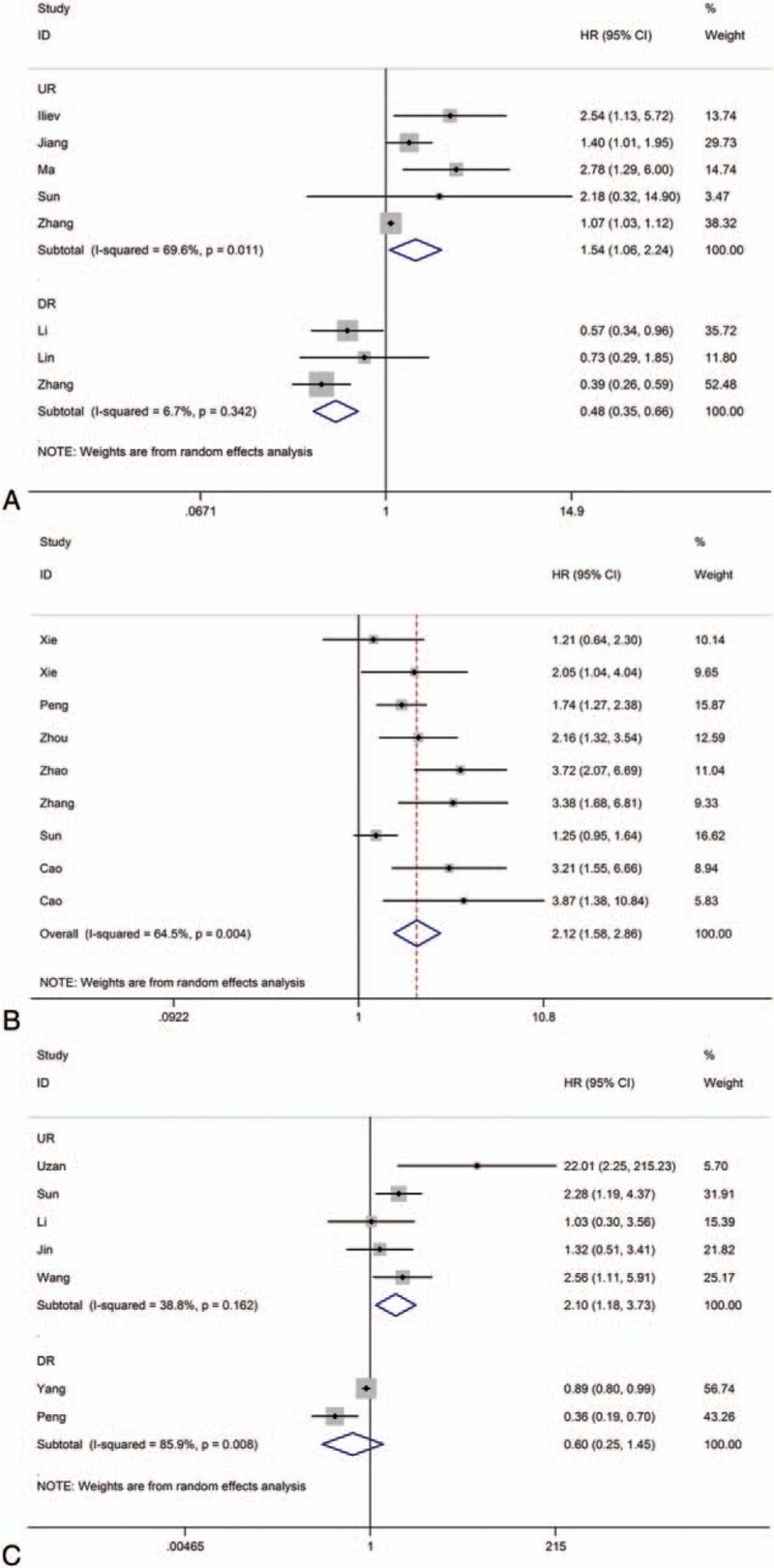
Forest plot of the correlation between 3 lncRNAs expression levels and OS in different cancer patients.

Eight of the included studies reported the overall survival (OS) of 962 patients according to TUG1 expression levels. The random-effects model that was used to calculate the pooled HR with corresponding 95% CI because the between-study heterogeneity among the upregulated group for TUG1 expression was confirmed (*P* = .011 for heterogeneity test, *I*^*2*^ = 69.6%). However, the significant heterogeneity did not exist across studies in the down-regulated group of 3 studies. The fixed-effects model was used to calculate the pooled HR with corresponding 95% CI for TUG1 expression was confirmed (*P* = .342 for heterogeneity test, *I*^*2*^ = 6.7%). According to meta-analysis result, it can be found that the expression of TUG1 might be associated with poor overall survival in different cancers. (Fig. 3A)

All of the included studies reported the OS of 962 patients according to SPRY4-IT1 expression levels. For evaluating the association between SPRY4-IT1 expression and prognosis more reasonably, the random-effect model that was used to calculate the pooled HR with corresponding 95% CI because the between-study heterogeneity among studies SPRY4-IT1 expression was confirmed (*P* = .004 for heterogeneity test, *I*^*2*^ = 64.5%). According to meta-analysis result, it is known that high expression of SPRY4-IT1 might be associated with poor overall survival in tumors (pooled HR = 2.12, 95% CI 1.58–2.86, *P* < .0001) (Fig. 3 B). In a word, the cancer patients with high expression of SPRY4-IT1 might be correlated with bad prognosis.

Seven included studies reported a total of 1037 patients with OS according to HULC expression levels. The fixed-effects model that was used to calculate the pooled HR with corresponding 95% CI because the between-study heterogeneity among the upregulated group for HULC expression was confirmed (*P* = .162 for heterogeneity test, *I*^*2*^ = 38.8%). However, the significant heterogeneity did exist across studies in the other group of 2 studies. The random-effects model that was used to calculate the pooled HR with corresponding 95% CI for HULC expression was confirmed (*P* = .008 for heterogeneity test, *I*^*2*^ = 85.9%). According to meta-analysis result, it can be seen that the expression of HULC might be associated with poor overall survival in various types of carcinomas (Fig. 3C). All the meta-analysis results were summarized in Table 3.

**Table 3:**
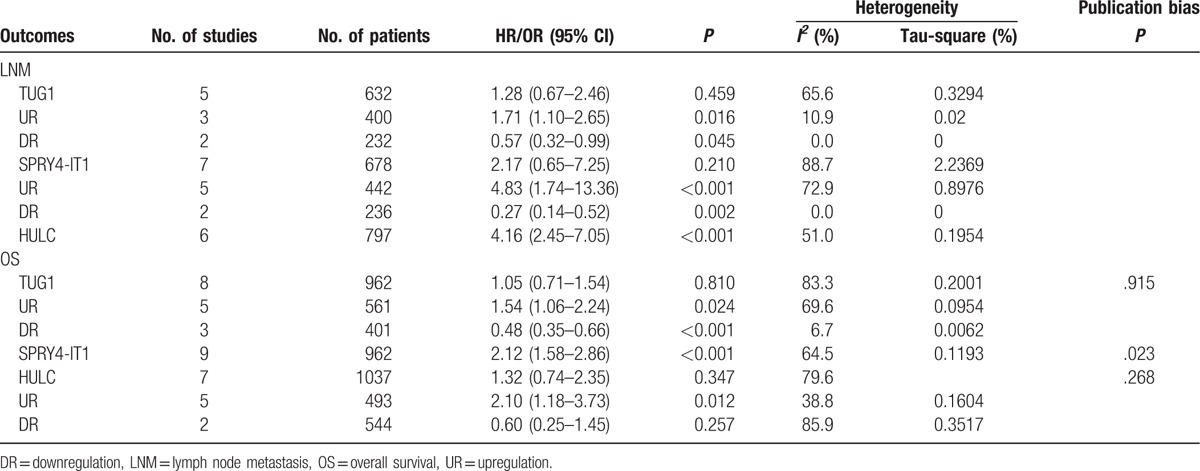
Results of this meta-analysis.

### 3.4 Publication bias and Sensitivity analysis

Publication bias of the present meta-analysis was evaluated by Egger test. In OS group, according to Egger's test (t = 2.90, *P* = .023), publication bias was shown in group SPRY4-IT1, whereas no significant publication bias was observed by the Egger test in the other 2 groups (Supplement-1). Sensitivity analyses were presented in Supplement-2. Although a single study each time in 3 groups was removed, there was no significant impact on the result patterns.

## 4 Discussion

The more we learnt about lncRNAs, the more awareness we got that lncRNAs expression might predict poor OS in cancer patients. However, what methods should be taken to summarize the results of these experiments? In clinic, meta-analysis is a commonly used research tool. Such analysis can summarize all the similar researches and provide a direction in clinical work. However, the concept of combining meta-analysis is not easy; both statistical and biological analyzes are required. It is different from basic research for it is not a simple combination of all outcomes, but understanding and dealing of the intricate results with professional thinking, even sometimes the evidences are conflicting, and it can improve our comprehension of biological systems. This is the first meta-analysis to evaluate the association between 3 well-known lncRNAs levels and clinical prognosis about cancer. The current meta-analysis has been conducted to explore the correction between expression levels of these 3 lncRNAs and overall survival rate for cancer patients. Our results are shown in Table 3, which demonstrated that the expression of TUG1, SPRY4-IT1 and HULC could predict poor survival in diverse types of cancers for patients. Through the above analysis, it can be seen that TUG1, SPRY4-IT1, and HULC were novel predictive factor of poor prognosis in most cancers. Meanwhile, these studies indicated that a signaling pathway can cause extracellular signaling molecules entering into the cell and can directly affect the phenotype of cells, such as cell proliferation, apoptosis and invasion and metabolism. To further study the mechanisms of cancer and targeted therapy and explore the significance of these 3 lncRNAs, a review of TUG1, SPRY4-IT1, and HULC including their potential targets, pathways and related miRNA in this meta-analysis has been systematically made (Table 4).^[21,23,25,36–79]^

**Table 4:**
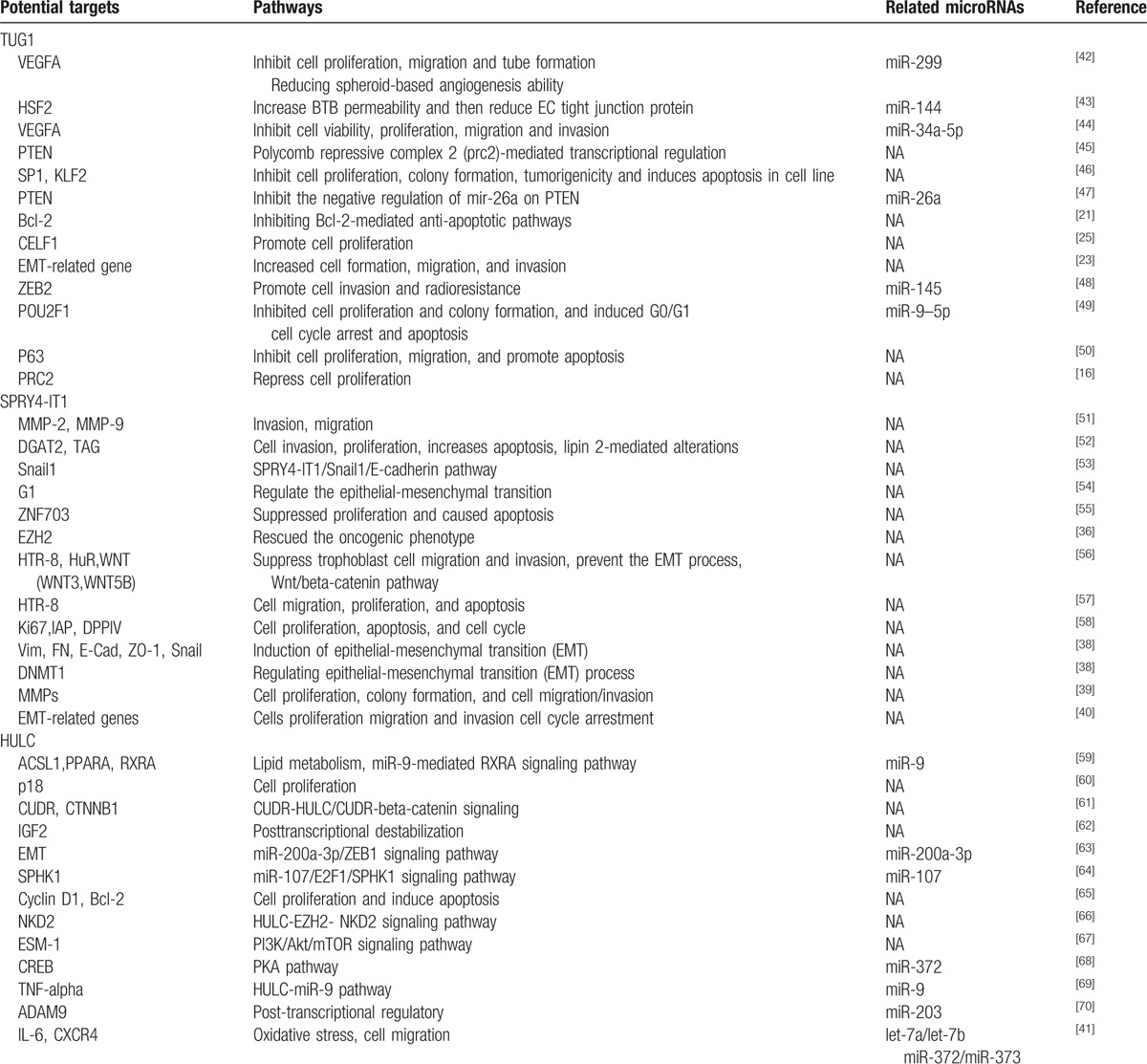
Summary of 3 lncRNAs with their potential targets, pathways and related microRNAs entered this study.

## 5 Limitations

However, it should be recognized that there are still several limitations in the current meta-analysis. First, given that this report is based only on the results of four databases (PubMed, EMBASE, Cochrane Library, and Web of Science), it is possible that some relevant papers have been missed out. Second, the cut-off definition of lncRNAs expression was different in each study because it was difficult to define a standard cut-off in different types of cancers. Third, some of retrieved articles may not have provided the most accurate estimate of the HR as much as possible, because these data were extracted from Kaplan–Meier curves at most times. However, this approach does not produce a significantly different result from the direct method of HR estimation. Fourth, tau-square, alike *I*^*2*^, is largely affected by the size of the studies and the number of the included studies. As a result, it may be very misleading in our moderately sized meta-analysis. Until now it is still not a good way to deal with the issue of heterogeneity. Fifth, the samples of the study are limited. Only 26 studies were totally included in the current meta-analysis, which might weaken the reliability of this current meta-analysis’ consequences. Sixth, most of the included studies reported that lncRNAs were overexpressed in different types of cancers; low expression of lncRNAs studies was generally less likely to be published. Thus, the results of this meta-analysis should be upheld by future studies.

Despite of the significant progress in early diagnosis, surgical techniques, and chemotherapy, the prognosis of patients with cancer is yet unsatisfactory. There are still many challenges to be dealt with, for example, lack of diagnostic and prognostic markers for cancers, limited efficiency treatment, and molecular targeted therapy. Therefore, more and more novel strategies should focus on the identification of innovative prognostic biomarkers of cancers. In recent years, lncRNAs were found out relating with human cancers and more than thousands of lncRNAs have been found and reported, such as AFAP1-AS1,^[80]^ H19,^[81]^ UCA1,^[82]^and HOTAIR.^[83]^ More and more reports point out that lncRNAs could act as tumor markers in both diagnosis and predicting the prognosis.^[84]^ Currently, however, there are a few meta-analyses to summarize the study of lncRNAs and molecular markers in prognosis of cancer. There is no doubt that lncRNAs are important regulators in various types of human cancers,^[85]^ thus, the research is promising in the area of tumors.

## 6 Conclusions

In conclusion, this meta-analysis provides evidences that expressions of 3 lncRNAs (TUG1, SPRY4-IT1, and HULC) are significantly associated with overall survival in cancer patients, which means that these 3 lncRNAs may have great potential to be an accurate biomarker to reveal the value of diagnosis and prognosis in diverse cancers. However, there are still many difficulties to overcome, more and more well-designed studies according to the PICOS setting and methodological characteristics (eg, randomization, blinding) with large sample sizes are required to confirm the validity and effectiveness of applying TUG1, SPRY4-IT1, and HULC in the diagnosis and prognosis of cancer patients.

## Acknowledgments

The authors are indebted to the donors whose names were not included in the author list, but participated in this program.

## Supplementary Material

Supplemental Digital Content
