# Supplementary material for: TUG1, SPRY4-IT1, and HULC as valuable prognostic biomarkers of survival in cancer: A PRISMA-compliant meta-analysis
Source: Medicine (Baltimore). 2017 Nov 17;96(46):e8583. doi: 10.1097/MD.0000000000008583 (PMC5704816; doi:10.1097/MD.0000000000008583)

Supplement.1 Funnel plot analysis of potential publication bias in OS group (Egger’s test).


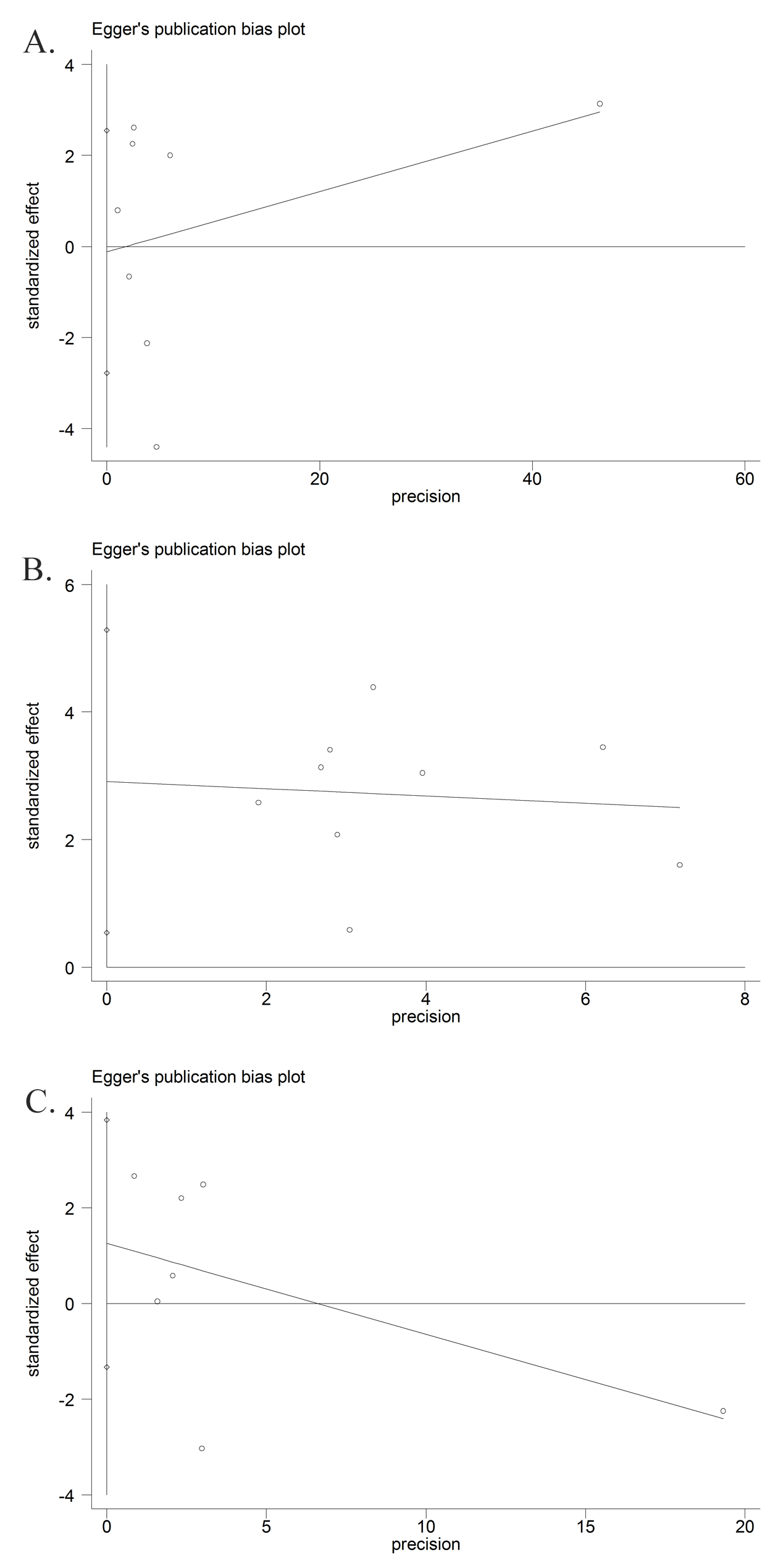


Supplement.2 Sensitivity analysis of effect of individual studies on the pooled HRs for 3 lncRNAs and overall survival of patients.


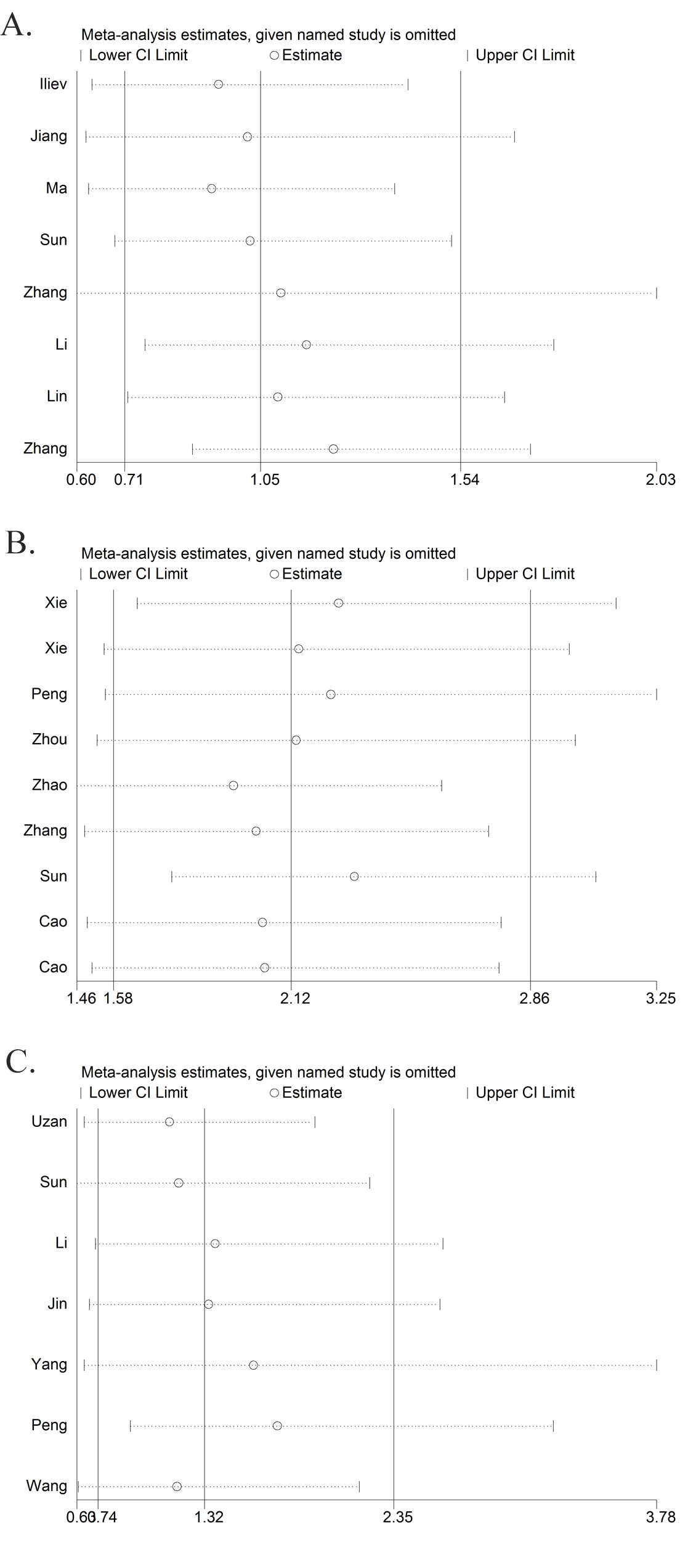

Supplement: Supplemental Digital Content [file medi-96-e8583-s001.doc]
